# Feasibility and Form Factor Validation of Reflective Shoulder-Mounted Pulse Oximeter in Patients with Suspected Sleep Apnea

**DOI:** 10.3390/s26041276

**Published:** 2026-02-15

**Authors:** Katie N. Kanter, Aaron Wang, David Gordon, Adina Singer, Jacob S. Brenner, Indira Gurubhagavatula, Anush Lingamoorthy, Olumuyiwa Oni, Cameron M. Baston

**Affiliations:** 1 Perelman School of Medicine, University of Pennsylvania, 3400 Civic Center Blvd, Philadelphia, PA 19104, USA; 2 Sidney Kimmel Medical College, 1025 Walnut St #100, Philadelphia, PA 19107, USA; 3 College of Electrical Engineering, Drexel University, 3141 Chestnut St, Philadelphia, PA 19104, USA

**Keywords:** pulse oximetry, wearable biosensors, user acceptance, sleep apnea, respiratory monitoring

## Abstract

**Highlights:**

**What are the main findings?**

- Shoulder-based pulse oximetry results in decreased noisy data compared with traditional finger-based pulse oximetry.
- Shoulder-based pulse oximetry is more comfortable than traditional finger-based pulse oximetry, resulting in fewer device removals and decreased self-reported sleep disruption.

**What is the implication of the main finding?**

- New medical devices targeting the shoulder for respiratory monitoring in sleep-disordered breathing patients will be well received by patients.
- It is feasible to get a high accuracy from shoulder-mounted devices.

**Abstract:**

The shoulder may be an effective central site for continuous oxygen saturation (SpO2) monitoring but studies of shoulder-mounted pulse oximetry technology are limited. We hypothesized that an alternative location would be similar in function and user acceptance to a standard FDA-cleared finger-based pulse oximeter. We conducted a quantitative and descriptive pilot study of two prototype biosensor designs in patients with clinical suspicion of hypoxic episodes at an outpatient sleep center. Participants wore two prototype biosensors—the primary a shoulder-mounted adhesive and the secondary a combination ring–bracelet—in addition to a control FDA-approved finger-based pulse oximeter. We assessed the comfort of the devices based on a survey. We monitored 27 patients during an overnight polysomnography study. Participants rated the shoulder-mounted device more highly than the control device on a Likert scale survey of comfort (4.6 out of 5 versus 3.1 out of 5). Open-ended questionnaires showed that the two major criticisms of the control and ring devices were devices falling off and disruption to sleep, while only one participant commented on the shoulder device specifically. We also investigated SpO2 agreement between the primary shoulder-mounted prototype and the control finger-based pulse oximeter. This study confirms that alternative configurations for SpO2 monitoring offer potential as well-tolerated devices with preliminary findings of acceptable agreement. Problems with traditional pulse oximetry, such as false readings of hypoxia due to device removal or noisy data, were encountered less frequently in shoulder-mounted pulse oximetry than in the commercial finger-based device. Future directions include studies of additional populations that are at risk of respiratory collapse and surveys to elicit specific feedback on the configurations, whether positive or negative.

## 1. Introduction

Wearable continuous monitoring technologies are increasingly common in healthcare [1,2]. Continuous pulse oximetry would benefit several high-risk populations including patients with chronic lung disease, sleep-disordered breathing, and patients with active opioid use [3,4,5]. Conventional pulse oximeters limit the use of the hands, but alternate pulse oximeter probe sites range from the forehead to the toes, sometimes with smartphone interfacing [6,7,8,9,10]. Motion artifacts in ambulatory patients limit accuracy, potentially resulting in the delayed detection of hypoxemia [1,11]. Wearable biosensor technologies in the outpatient setting must be designed for user acceptability and device effectiveness and hold the potential for additional features such as integrated respiratory monitoring [4,12,13]. Finger-based pulse oximeters are particularly vulnerable to signal dropout and false desaturation during sleep due to motion, peripheral vasoconstriction, and inadvertent device removal given their position on the highly innervated fingertip.

Studies of shoulder-mounted pulse oximetry technology are limited [14,15]. Our group developed a shoulder-mounted device with a high level of acceptability in certain populations [16]. We sought to investigate the feasibility and acceptability of a shoulder-based pulse oximeter among a convenience cohort undergoing diagnostic polysomnography.

## 2. Materials and Methods

We conducted a quantitative and descriptive pilot of two prototype biosensor designs at the Penn Sleep Medicine Diagnostic Program, an outpatient sleep center in Philadelphia, PA, USA. The study protocol was approved by the University of Pennsylvania IRB and was performed in compliance with relevant laws and institutional guidelines. Inclusion criteria included clinical suspicion for sleep-disordered breathing and age > 21. Exclusion criteria included pregnancy.

**Figure 1:**
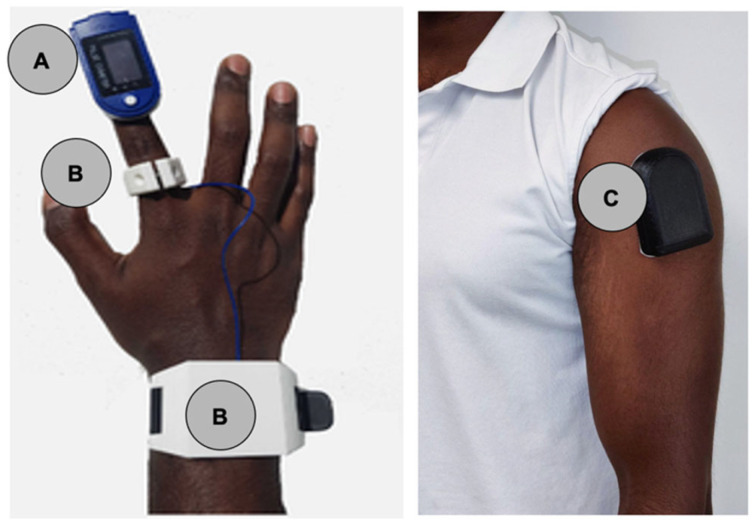
(**A**) Commercial pulse oximeter control; (**B**) Prototype-ring configuration; (**C**) Prototype-shoulder configuration.

Patients wore a standard pulse oximeter EMO-80 Sleep Oxygen Monitor, (EMAY Hong Kong, China, Figure 1A) and two prototype biosensors: Prototype-ring (Figure 1B) and Prototype-shoulder (Figure 1C). Prototype-ring (Figure 1B) consisted of a ring worn on the index finger to assess oxygen saturation as well as a wristband containing a battery and data storage hardware. Prototype-shoulder (Figure 1C) was an adhesive armband worn over the deltoid. Prototype-shoulder was designated the primary investigational device for performance and agreement analyses; Prototype-ring was included for exploratory comparison and user perception assessment.

We assessed the prototypes based on comfort via a 5-point Likert scale and an open-ended qualitative questionnaire. In addition, we compared the calculated SpO2 of Prototype-shoulder to the FDA-approved fingertip sensor as per the FDA guidelines for pulse oximeters [17]. We also evaluated independent patient-level variables including demographics and participant BMI. Differences in mean results were assessed with a two-sided *t*-test with alpha 0.05.

The Prototype-shoulder device employs reflective optical spectroscopy to non-invasively gather photoplethysmography (PPG) data directly from the skin of the upper arm. We computed the conventional ratio-of-ratios (ratio of modulation) RSpO2 from the red/IR AC and DC components and included it as an input feature to the ML model. Then the model was trained on the entire dataset using a 70-15-15 split (train–test–validate) using SpO2 labels from the commercial finger pulse oximeter. The dual-channel optical sensor employs red and infrared (IR) LEDs with wavelengths of 660 nm and 880 nm, respectively, to interrogate dermal and subdermal vascular beds over the deltoid region. PPG signals were sampled at 25 Hz and stored locally for offline processing. The device enclosure was designed to minimize ambient light interference while maintaining patient comfort during sleep. The raw PPG and motion data were preprocessed using a Chebyshev Type II bandpass filter to mitigate noise; it operates between 0.5 Hz and 3 Hz for SpO2 and pulse rate monitoring. We trained the model using an 80-20 train–test split on a 5-fold cross validation. We then tested the R2 score, mean absolute error, and standard deviation of the mean absolute error to ensure the model was not overfitted. No adjustments were made for known confounders such as skin tone, positioning, or sleep stage.

Specifically, we textually summarize the downsampling pipeline as follows: Raw data were preprocessed for smoothness, calculating the relative magnitude of motion (*R_motion_* = $\sqrt{x_{axis}^{2}+y_{axis}^{2}+z_{axis}^{2}}$) and removing values for which 0.8 < *R_motion_* < 1.2. Processed data was filtered to have values only between SpO2 99 and 88% as these were the values of interest (with anything below 88% being a clinical event). Each SpO2 value was binned to identify the lowest SpO2 bin count, based on which all SpO2 values were randomly decreased. This ensured a uniform distribution and decreasing bias when training the model. Using the new downsampled uniform distribution, we passed it through our model using an 80-20 train–test split on a 5-fold cross-validation. Overall, 5 models were generated and 20% of data was used as the test data. Based on the R2 score, mean absolute error, and standard deviation of the MAE, the model did not overfit or underfit. It was trained following all the standard practices for a 5-fold cross-validation.

## 3. Results

### Study Participants

**Figure 2:**
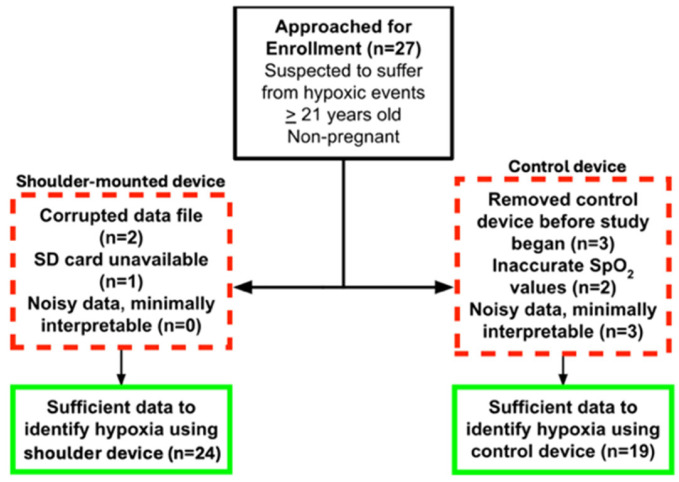
Proportion of devices with sufficient data to identify hypoxia. Red dashed boxes demonstrate patients lost from hypoxia detection analysis.

Participant demographic information is described in Table 1. The patients sampled were majority white (57.1%) and male (58.3%). Participants had a median BMI of 28.5 (IQR 26.5–35.1). A consort diagram is shown in Figure 2. Data from the shoulder was unusable for three patients due to corrupted data files (two) and SD card unavailability (one). The FDA-cleared control device had eight patients with unusable data. All patients with interpretable finger pulse oximetry data had interpretable shoulder-mounted data.

**Figure 3:**
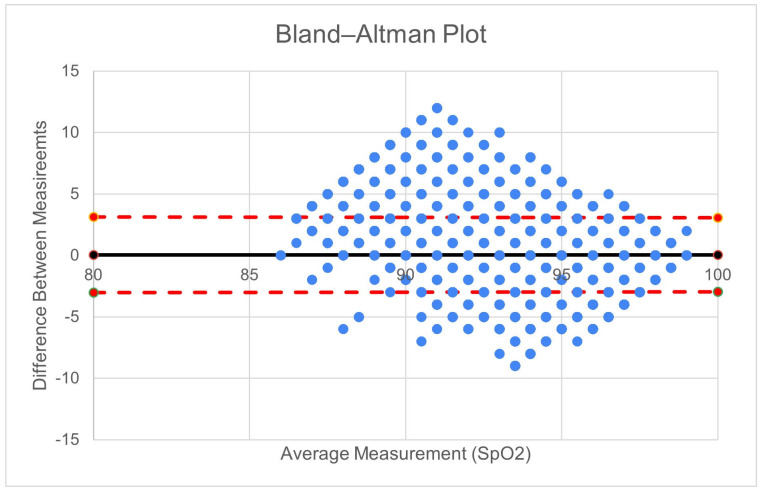
Bland–Altman plot showing agreement between the commercial pulse oximeter to measured SpO2 with Prototype-shoulder for 354,002 measurements. Red dashed lines indicate 95% confidence intervals of agreement.

**Figure 4:**
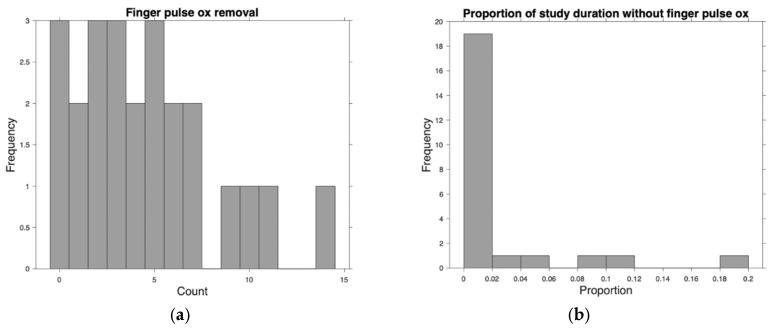
(**a**) Number of commercial pulse oximeter removals. (**b**) Proportion of study duration during which the finger pulse oximeter was removed.

**Figure 5:**
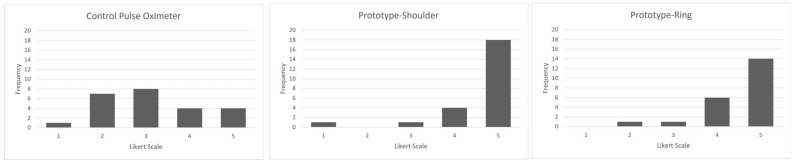
Quantitative Likert scale feedback regarding the comfort of the study-provided devices: 1 is very uncomfortable, 5 is very comfortable.

Agreement among the 354,002 measurements made on 19 patients with data from both devices is shown in Figure 3. The Prototype-shoulder pulse oximeter had a 0.72% mean absolute error from the control values of the commercial finger-based pulse oximeter. Participants removed the prototype 0 times and the control device a mean 4.6 (sd 3.7) times (Figure 4a)—the time removed is shown in Figure 4b. The five-point Likert scale feedback on comfort showed a mean rating of 4.6 for the shoulder, 4.5 for the ring, and 3.1 for the finger-mounted control (Figure 5). Both the shoulder and ring were rated higher than the control device (*p* < 0.01).

Themes were identified in open-ended questions regarding the comfort or fit of the study-provided devices: Many participants reported that their control devices fell off either before falling asleep or during the night. One participant noted that the finger-based “commercial device came off a few times before sleeping” which caused “restless sleep”. Several participants also noted that the control devices either fell off or were purposefully removed throughout the night. Participants cited irritation, fit, and using the restroom as reasons for control device removal. One person noted that the wristband portion of the Prototype-ring fell off in the middle of the night. None complained of comfort worsened by the shoulder-mounted device. Several participants complained that one or multiple devices disrupted their sleep. These devices included the Prototype-ring and commercial pulse oximeter. Tightness of fit was the most commonly problematic factor among these devices. One participant specifically cited the plastic shell that encased the data hardware of Prototype-ring as causing pain. Other participants removed the control device due to discomfort of a “burning sensation” or complaints that the device “was getting hot.” There was only one participant who specifically commented on Prototype-shoulder. This person stated that they slept on their side but there was “not much movement [to the device] while sleeping.”

## 4. Discussion

This study demonstrated that a shoulder-based investigational SpO2 monitor prototype was described as superior in comfort to a traditional commercial finger pulse oximeter. The open-ended questionnaire revealed that this stemmed from issues with security to the finger, fit, and temperature. Additionally, both shoulder-based and ring devices measured SpO2 with fewer interruptions to continuous monitoring than a traditional commercial finger pulse oximeter. Participants reported the accidental or purposeful removal of the commercial device both before and during their sleep studies, as well as interference with sleep from the control and ring devices. By contrast, the shoulder prototype did not produce the same reports. Additionally, the pulse oximetry performance of the shoulder-mounted device demonstrated acceptable agreement in this feasibility cohort. Collectively, these results support the concept that a shoulder-mounted pulse oximeter is a viable and potentially preferable configuration for SpO2 monitoring in patients receiving polysomnagraphy.

Shoulder-mounted pulse oximetry is also pertinent as an alternative to current finger-based wearables because it offers the opportunity to combine SpO2 monitoring with additional features, such as accelerometry, to detect apneic motion [4,12,15]. These applications are important for people with sleep-disordered breathing at the highest risk of respiratory compromise [13,18]. This study demonstrates that shoulder-mounted pulse oximeters may be more comfortable and less disruptive than traditional finger-based pulse oximetry. This could also be used to improve the overall accuracy of the device (see Supplemental Figure S1) in order to identify when motion artifacts are affecting sensitivity or specificity.

There were multiple limitations to this pilot. Firstly, the control pulse oximeter is an imperfect gold standard as its accuracy may be limited. Secondly, most participants did not provide constructive qualitative feedback for all study-provided devices, so it is difficult to evaluate the characteristics that drove improvements in Likert scale score. Additionally, we did not collect sufficient data to correlate data quality with the participants’ sleep position. While participants were able to recollect the removal and/or repositioning of the study-provided devices, the accuracy of their subjective reports is limited in the absence of a real-time record of behavior from the center’s technicians. Formal PPG signal quality indices, such as those proposed by Elgendi, were not applied in this pilot study [19]. Future work will incorporate established signal quality indices to enable the standardized assessment of signal robustness across anatomical sites. Finally, this study was not powered or designed to assess meaningful clinical outcomes, nor for generalizability to more ill populations.

## 5. Conclusions

Overall, this study confirms that alternative configurations for SpO2 monitoring offer potential as accurate and well-tolerated devices. Problems with traditional pulse oximetry, such as false readings of hypoxia due to device removal or noisy data, were encountered less frequently in Prototype-shoulder than in the commercial finger-based device. Users not only tolerated the shoulder-based form factor but also preferred this configuration relative to the traditional finger pulse oximeter.

## Figures and Tables

**Table 1:** Patient demographic information for the 27 recruited patients.

Age [median (IQR)]	55 (38.5–69.5)
Male (%)	58.3
Race (%)	
White	59.3
Black	29.6
Asian	7.4
Native Indian/Native Hawaiian/Other	0
BMI [median (IQR)]	28.5 (26.5, 35.1)

## Data Availability

Anonymized analyzed datasets are available on request.

## Supplementary Materials

The following supporting information can be downloaded at: https://www.mdpi.com/article/10.3390/s26041276/s1, Figure S1: Sample of motion vs SpO2 over time.

## Abbreviation

The following abbreviation is used in this manuscript:

SpO2	Peripheral Saturation of Oxygen
