# Supplementary material for: Feasibility and Form Factor Validation of Reflective Shoulder-Mounted Pulse Oximeter in Patients with Suspected Sleep Apnea
Source: Sensors (Basel). 2026 Feb 15;26(4):1276. doi: 10.3390/s26041276 (PMC12944660; doi:10.3390/s26041276)
Supplement: Supplementary file 1 [file sensors-26-01276-s001.zip › sensors-4077504-supplementary.pdf]

sensors

MDPI

Supplemental Material

Shoulder-based Accelerometer

The shoulder-mounted accelerometer was able to detect subject movement in three planes of motion. This represents information not provided by the commercial device, and was able to demonstrate that periods of relative hypoxemia on the commercial pulse oximeter were often associated with increased movement throughout the night.

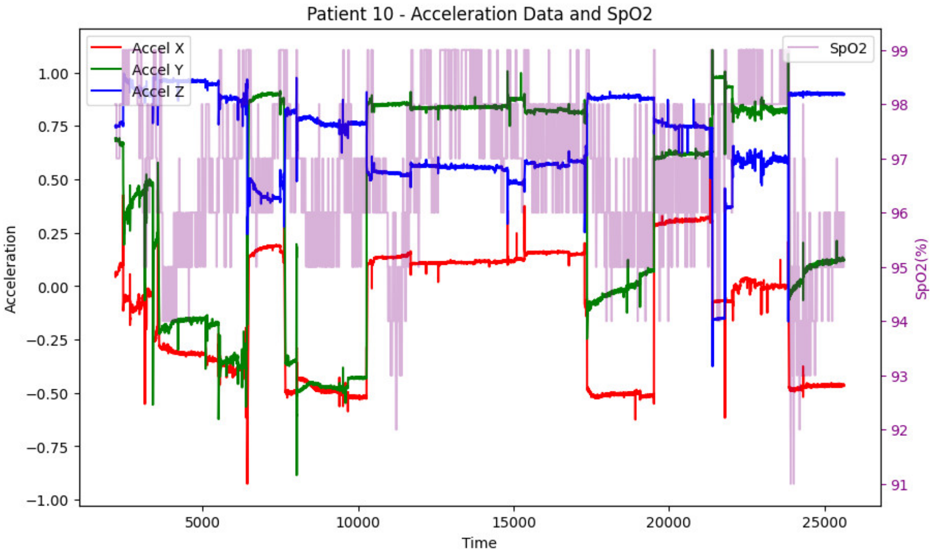

Supplemental Figure S1. Sample of Motion vs SpO_2 Over Time
